# Lectures on the Principles and Practice of Physic, Delivered at King's College, London

**Published:** 1844-01

**Authors:** 


### Art. VI.

*Lectures on the Principles and Practice of Physic, delivered at King's College, London.* By Thomas Watson, m.d., Physician to the Middlesex Hospital. In Two Vols.—*London,* 1843. 8vo, pp. 830, 812.

In rising from the perusal of this work,—for large as it is and recent as is its publication, we have read the greater part of it,—we feel irresistibly tempted to follow the example of certain reviewers, vulgarly denominated of the "Gutting School," and transfer the greater portion of it to our own pages. By this proceeding we should certainly achieve several objects which the gentlemen of this school regard as of paramount importance. In the first place, we should save ourselves a vast deal of trouble; as, instead of having to study the subject of the book both in itself and in others of the same kind, and thence to educe at once a true representation of the actual state of the things under discussion, and a critical judgment of the form in which they come before us,—we should merely, in skipping over the pages, have to mark in pencil all the passages to be extracted, and then get these orient pearls, strung at random, on the slenderest possible thread of our own manufacture. Secondly, we should save ourselves not a little money: as, in a manufactory of this sort, no other contributors but ourselves, the scissors, and the printer being requisite, we should have none of those compliments to pay at the end of the quarter which we feel it our duty to gently force upon our friends, as being the only means we know of securing our own independence and usefulness. Thirdly, we should add vastly to our own comfort and pleasure; as it would surely be much more agreeable to dig, quarter after quarter, for a year or two, out of Dr. Watson's gold and silver mines, or to heap our measures from his redundant granaries, than to torment our spirit in everlasting research after hidden treasures throughout the world; pounding the obdurate rocks into barren dust; or wearying to death our poor critical microcosm in first threshing the ears from the mountains of straw and then winnowing the grains from the hills of chaff. And, lastly, should we not thereby entitle ourselves to the gratitude of our readers, and increase the numbers thereof, by saving *their* purses also,—by giving them that for a few shillings which it might cost them many to get otherwise? To be sure the author might perchance complain that the condition of *his* purse was but little consulted in the matter; and that, while he was held up to the admiring world as the greatest of writers, he had to look elsewhere than to his publisher for the payment of his printer and paper-maker. But what of that? The "Gutting School" cannot swerve from its principles for considerations of a private nature; and the frequenters, and auditors, and readers of this school must not have their appetite baulked or their exchequer impoverished by any falling-off in their periodical supplies.

But as our hand is not yet well broken-in to this kind of work, we hope our readers will excuse the barrenness of our first essay; and allow us, before we commence our pearl-stringing, to say a few words, in our old fashion, respecting the general qualities and character of the *corpus delicti*, or subject to be gutted.

"The following lectures," says the author, in his modest advertisement, "were put together with unavoidable haste during the medical session of 1836-7, in which they were first delivered. They were repeated, with slight variations, for four successive years, the author always meditating but never finding time to accomplish their thorough reconstruction and revision. They were afterwards printed, to fulfil a rash promise, in the pages of the Medical Gazette; and they are now published in a collected form at the request, formally conveyed to him in writing, of many who had heard or read them, including many of his colleagues at King's College. . . . . As they were passing through the press, such additions and alterations have been introduced as the author would have made had he continued to deliver the lectures orally." (pp. iii-iv.)

The medical profession is under great obligations to the gentlemen, whoever they were, who induced the author to publish his lectures in their present form, as its members have thus obtained for their help and guidance a better and completer system of medical practice than they were before possessed of; and the medical literature of this country has been enriched with a work of standard excellence, which we can proudly hold up to our brethren of other countries as a representative of the actual state of British medicine as professed and practised by our most enlightened physicians. And, for our own parts, we are not only willing that our characters as scientific physicians and skilful practitioners may be deduced from the doctrines contained in this book, but we hesitate not to declare our belief, that for sound, trustworthy principles and substantial good practice, it cannot be paralleled by any similar production in any other country. And yet the author is very far from claiming any such character for his book; and we feel pretty certain, from the unpretending and modest way in which he expresses himself on all occasions when his own opinions are in question, that he does not believe it entitled to such honour. "The lectures," he says, "do not profess to present a formal and complete treatise on the practice of physic, much less to exhaust the various subjects upon which they touch. His chief hope is that they may prove a text-book for students."

That they will form an admirable text-book for students, in one sense, we do not doubt; but hardly in the common meaning of that phrase. The work is too large and too minute, in fact too good, for the use of the young student during his early attendance at the lecture-room and hospital. He requires something much more compendious; and we would be glad if Dr. Watson could find time to write such a book; he need not go one step beyond these lectures for materials for an admirable one. To the advanced student, however, and still more to the junior practitioner, these lectures will prove invaluable; and we would advise no one to set himself down in practice unprovided with a copy. Even among the old and experienced practitioners we venture to say there are few who will not benefit by a perusal of this work. Containing, as it does, all the more recent medical doctrines, without overlooking the old; combining the physiology, pathology, and morbid anatomy of recent times with the facts deduced from ancient observation; testing all theoretical views by the results of practical experience; weighing and checking everything—whether fact, doctrine, or opinion—with candour and calmness; and imbued throughout with that indefinable something which springs from strong common-sense;—it cannot fail to be acceptable and useful to every medical reader, whether young or old. The style of the lectures, too, is excellent. Simple, plain, and familiar, as the lecture-room required, they are, at the same time, most accurately and even beautifully written; combining the chaste graces of classical scholarship with the racy strength of our native Saxon. In the whole ninety lectures contained in these huge volumes, we have scarcely met with a single objectionable expression; while in every page we have been charmed with those nameless elegances which betray at once the native taste and the academic breeding of the writer.

Such of our readers as have observed how frequently we have felt ourselves called upon in this journal to expose and condemn the style of the medical writings that come before us, will not be surprised at this expression of our satisfaction with Dr. Watson's. And we may take occasion to add, that the volumes before us are yet more free from every tincture of those graver delinquencies which it has so often been our painful duty to expose. In Dr. Watson's pages the most critical eye will detect no defect of this kind. So far from any attempt to exalt, directly or indirectly, his own credit before his auditors, he never speaks of himself but with the greatest modesty; and never utters an expression calculated to depreciate others unjustly. At the same time, he delivers his own opinions with freedom, and his judgments with firmness; and never hesitates to condemn in others what he deems objectionable. But the hearers of Dr. Watson's lectures must have felt convinced that in him they had a professor no less qualified to be a guide in the path of pure professional morality, than an instructor in professional knowledge; and no one will rise from the perusal of them in their present shape without bearing away with him the gratifying conviction, that the author is as much a man of the strictest honour, as he is a most accomplished physician.

And now to our pearl-stringing.

The first lecture is introductory, giving a general view of the subjects to be treated of, and of the proposed method of treating them. It affords a good sample of what is to be expected in the book. It takes broad, comprehensive, common-sense views of the subject; without any useless refinement, any announcement of trifling theories, or any covert magnifying of the lecturer. He professes to teach only *the principles* of medicine:

"The *practice* of medicine, or the particular application of those general facts and doctrines, I shall describe to you, but I cannot profess to *teach* it in this room; nor can you learn it, except in a very imperfect sense, from my description of it. It is the *science* that I shall here endeavour to unfold. Skill and facility in turning that science to useful purposes I am unable to impart. These are qualities that do not admit of being communicated from one mind to another. The practice of physic, like every other practical art, is to be learned by its repeated exercise, by habit, by carrying its various acts into direct effect again and again, or if they happen to require no manual dexterity, by looking on, and seeing them done again and again. There is this capital difference, however, between the art of healing and some other arts: that the blunders of early attempts may be both grievous and irremediable—may hurt or spoil the goodly and precious machine they are intended to repair." (p. 10.)

The lecture concludes with the following beautiful exposition of the responsibilities and privileges of medical teachers and medical practitioners:

"The subjects with which we have to deal are not matters of mere speculative curiosity or intellectual amusement—to be taken up to-day and dismissed perhaps with unconcern to-morrow; but they involve questions of life and death. The opinions you are now to form or to embrace are, for the most part, the opinions upon which in after-life you will confidently and constantly be acting. The comforts or the misery of many families may probably hang upon the notions that each of you will carry from this place. Therefore it is that I feel myself to be engaged in a very serious undertaking. Doctrines and maxims, good or bad, flow abroad from a public teacher as from a fountain, and his lessons may become the indirect source of incalculable mischief and suffering to hundreds who have never even heard his name. These reflections fill my mind with an almost painful sense of the obligation imposed upon me, by my present office, of closely sifting the facts, and of carefully examining the principles to be derived from those facts, which I propose to employ for your instruction and guidance.

"But amid all the responsibilities, gentlemen, both as teacher and learner, the profession which you and I have chosen, or which circumstances have prescribed to us, is a noble profession, and worthy the devotion of a life-time. If you fit yourselves now for its high functions, and pursue it hereafter with earnestness and truth, it will probably conduct you to an honorable competence, and it will assuredly prove a salutary school of mental and moral discipline. Trials, no doubt, belong to it, and difficulties; but it has also privileges and immunities peculiar to itself. Affording ample scope and exercise for the intellect, it is conversant with objects that tend to elevate the thoughts, to chastise the feelings, and to touch the heart. I have already reminded you how it brings beneath our minute and daily notice that most remarkable portion of matter which is destined for a season to be the tabernacle of the human spirit, and which, apart from that singularly interesting thought, excites increasing wonder and admiration the more closely we investigate its marvellous construction. The sad varieties of human pain and weakness with which our daily vocation is familiar should rebuke our pride, while they quicken our charity. To us are intrusted, in more than ordinary measure, opportunities of doing good to our afflicted fellow-creatures—of showing love towards our neighbour. Let us beware how we idly neglect or selfishly abuse a stewardship so precious yet so weighty. The profession of medicine, having for its end the common good of mankind, knows nothing of national enmities, of political strife, of sectarian dissensions. Disease and pain the sole conditions of its ministry, it is disquieted by no misgivings concerning the justice and honesty of its clients' cause; but dispenses its peculiar benefits, without stint or scruple, to men of every country, and party, and rank and religion, and to men of no religion at all. And, like the quality of mercy, of which it is the favorite handmaid, 'it blesseth him that gives and him that takes;' reading continually to our own hearts and understandings the most impressive lessons, the most solemn warnings. It is ours to know in how many instances—forming indeed a vast majority of the whole—bodily suffering and sickness are the natural fruits of evil courses; of the sins of our fathers, of our own unbridled passions, of the malevolent spirit of others. We see, too, the uses of these judgments, which are mercifully designed to recall men from the strong allurements of vice and the slumber of temporal prosperity; teaching that it is good for us to be sometimes afflicted. Familiar with death in its manifold shapes, witnessing from day to day its sudden stroke, its slow but open siege, its secret and insidious approaches, we are not permitted to be unmindful that our own stay also is short and uncertain, our opportunity precarious, and our time, even when longest, but scanty, if measured by our moral wants and intellectual cravings." (pp. 14-15.)

The first sixteen lectures, extending over a space of about 270 pages, are devoted to the important subjects usually comprehended under the head of general pathology. They give an admirable view of the present state of our knowledge. The various subjects are treated in the same bold style, indicated in the introduction; without partiality or prejudice, and with a constant subordination of the scientific or theoretical part to the results of observation and experience. The eighth lecture is on semeiology, and is worthy of the particular notice of the student. We cannot resist the temptation of selecting a few pearls from it, for the adornment and enriching of our string:

"*Symptoms and signs.* These words are not exactly synonymous, although they are frequently employed as if they were so. Even those medical writers who admit a distinction between them, have not always succeeded in clearly pointing out the difference. Signs are deduced from symptoms by arranging and comparing them, and noticing the circumstances under which they occur. Symptoms are obvious to all persons alike—to the nurse as well as to the physician: signs, for the most part, are such to medical eyes alone. Let me try to make this plainer by the help of an illustration. Symptoms may be considered as resembling so many *words.* When taken separately or put together at random, the words have no force or signification. Arrange them in due order, reduce them into a sentence, and they convey a meaning. The sentence is a *sign* or expression of something which is thus revealed. Symptoms become signs when their import can be interpreted.

"A certain crackling sound, of which I shall have much to say hereafter, is heard (we will suppose,) in some part of a patient's lung, by the ear applied outside his thorax. This sound is a symptom; any one who listens may perceive it. It is even so far a *sign*, that it denotes the unnatural presence of a liquid in the lung, and the passage of air through that liquid. But the liquid may be one of several—mucus, or serum, or pus, or blood: we cannot tell by the sound alone which of these it is. But if we learn that the person in whose lung the sound is audible has been ill for a day or two only, that he has pain in his chest, cough, embarrassed breathing, and fever, we conclude that he is labouring under that serious disease, *inflammation* of the lung. The crackling sound alone could not assure us of this; nor without the addition of this sound could the pain, the laboured breathing, the cough, or the fever. Taken collectively, the symptoms constitute a *diagnostic sign*, and bespeak the existence of pneumonia." (pp. 115-16.)

The remarks on diagnosis and prognosis are excellent. We regret that our extracts must be so limited:

"*Diagnosis*. When we can once identify a given diseased condition, we obtain the privilege of watching the behaviour of that diseased condition, again and again, under the operation of therapeutic measures; and from that time the increase of our knowledge concerning the appropriate management of that particular disease becomes progressive and sure. The term experience is obviously misapplied, and the results of all observation are vitiated when any doubt exists about the sameness of the objects contemplated. It is mainly to this imperfection in the diagnostic part of medicine that we must attribute the uncertainty and variation both of doctrine and practice which have brought so much suspicion, and reproach and ridicule upon the science we profess. *False* experience, if I may use such a term, has greatly hindered the progress of the healing art; and *false experience* springs from *false diagnosis*. A man will tell you that he has cured a score of cases of advanced phthisis; but he has deceived himself: they were not cases of true phthisis, but simply cases of chronic inflammation, with puriform discharge of the mucous membrane of the bronchi. He publishes an account of his success, and of his plan of treatment; and thus he deceives others also; and thus he retards the science which he fondly and conscientiously believes he is promoting. Accuracy of diagnosis, therefore, cannot be too highly estimated, nor too diligently sought after." (pp. 112-13.)

Under the head of *prognosis* we have the following very sensible observations, which we quote for the especial benefit of young practitioners. By following the judicious advice here given, they may escape with credit from many perplexing and difficult dilemmas:

"There is always some risk of losing as well as of gaining credit by strong statements and predictions of the death or the recovery of a patient. If you give an unfavorable prognosis you have a good chance of losing your patient altogether; his friends argue very naturally, that you are not infallible, that you may be wrong, that if *you* know of no means of safety for him some other practitioner may, and they *will* grasp at whatever straw comes near them. Do not suppose that this is merely a selfish view of the matter: it is often of much moment to the patient himself that he should not be tempted to put his life under the charge of impostors, who will feed his hopes and promise largely, and torture him perhaps with their discipline, and have no mercy upon his pocket. Many an instance have I known of persons dying of consumption, who, when given over by their regular attendants, have been brought to London at considerable expense, to exchange the many comforts of home for the inconveniences of a hired lodging, that they might be *cured* by that ignorant, and cruel, and rapacious quack, Mr. St. John Long. There are other reasons, too, why we must sometimes conceal the truth from our patients. It often happens that a person is extremely ill and in great danger, but yet may recover if he is not informed of his peril. To tell a person in these circumstances that he is likely to die, is to destroy his *chance* of recovery. You kill him if you take away his hope of living. It must be confessed that the duty of the medical man in these cases is very painful and embarrassing: the patient and patient's friends are urgently inquisitive to know whether there is any danger, or whether he is not yet out of danger. The rule which I have always adopted in circumstances of this distressing kind, when I see clearly that the case is hopeless of cure, is to fix as well as I can upon that person among the family or friends of the patient to whose prudence the real state of the matter may be the most safely confided. If I think that there is a possible chance of recovery, and that a knowledge of his danger by the patient would diminish that chance, of course I urge the necessity of speaking to *him* with assumed cheerfulness and confidence; if I see that the case is absolutely and inevitably mortal, either soon or at some little distance of time, I leave it to the discretion of the person with whom I communicate to disclose or conceal my opinion, as he or she may think best." (p. 114.)

Six lectures are devoted to the consideration of the important subject of inflammation, in its various forms and consequences: they are full of most valuable knowledge, both pathological and practical. We regret to let them pass without paying toll; and yet we hardly know what to select. The fourteenth lecture, on the *treatment* of inflammation, is particularly interesting; and we will extract a small part of what is said on the use and abuse of mercury:

"*Curative effects of mercury.* Next to bloodletting, as a *remedy*, and of vastly superior value, upon the whole, to purgation, in serious inflammations of various kinds, is *mercury*. This mineral is really a very powerful agent in controlling inflammation, especially acute, phlegmonous, adhesive inflammation, such as glues parts together and spoils the texture of organs. . . . . . . . In common adhesive inflammation, whether of the serous or the cellular tissues; whenever, in fact, you have reason to suppose that coagulable lymph is effused, or about to be effused, and mischief is likely to result from its presence, then you may expect much benefit from the proper administration of mercury; as an auxiliary, however, to bloodletting, not as a substitute for it. On the other hand, mercury is likely to be hurtful in those forms of disease " where the morbid action approximates to its own action." In cases of erysipelatous inflammation having a disposition to gangrene; in scrofulous diseases; in inflammatory complaints attended with general debility, and an irritable condition of the nervous system, or a manifest tendency to take on a typhoid character."

"*Mode of action.* If we inquire what mercury does when it is administered to a person in health, we find three very marked effects following its internal use. They vary, indeed, in different cases and under different circumstances; but we know that the employment of mercury under any of its usual forms of exhibition is often followed by increased watery evacuations from the intestines; or by an increased discharge of bile; or by an increased flow of saliva; that is to say, it determines (as the phrase is,) to certain secreting organs—the mucous membrane of the bowels, the liver, the salivary glands; it augments their natural secretion? and in this augmentation of secretion is implied an increased afflux of blood to the secreting part. It is probable that mercury has a similar influence on most or all the secreting surfaces of the body, altering the condition of the capillary circulation throughout. And an explanation of its curative power in inflammation has been drawn from this fact: it has been supposed that mercury thus tends to *equalize* the circulation; that by causing the blood to be distributed in larger quantity than common upon *several* surfaces at the same time, it obviates *pro tanto*, its excessive congestion or accumulation in any one organ. Whether this hypothesis in respect to the *modus operandi* of mercury be true or not, I will not pretend to say; but it certainly is not an unreasonable hypothesis." (pp. 228-30.)

*Treatment of mercurial sore mouth.* There are few accidents more provoking to the young practitioner than the not uncommon, undesired, and unexpected supervention of profuse salivation during the exhibition of mercury. We fear all the comfort we can give him in such a case is contained in the following extract. Like Dr. Watson, we have heard much of antidotes and potent expedients for speedy relief, but we never yet met with them in reality.

"You will constantly be called upon to do something for the relief of this disease (for so we must call it), which you yourselves, or some of your brethren, have with the best intentions inflicted. I have tried all sorts of expedients; and I have asked a great number of my friends what is the best plan to adopt in such cases: but I never could get much satisfactory information from them. Some thought purging was the best thing. Others recommended alum gargles, or gargles made with the chloride of soda; and these last certainly have one good effect, that of correcting the fetor. A dilute solution of chlorine in water, much used at the Middlesex Hospital, is better still. Others believed that sulphur, which has long been prescribed in such emergencies, was really of service; and some advised that the patient should be as much as possible in the open air: a few commended iodine. All admitted that they knew of no certain remedy. Neither do I. But there are two or three expedients which I am confident are often of very great use in checking the violence of the salivation, and in removing the most distressing of its accompaniments. If there be much external swelling, treat the case as being, what it really is, a case of *local inflammation:* apply eight or ten leeches beneath the edges of the jaw bones, and wrap a soft poultice round the neck, into which the orifices made by the leeches may bleed; and I can promise you that, in nine cases out of ten, you will receive the thanks of your patient for the great comfort this measure has afforded him. Pure tannin, moistened and smeared upon the spongy gums, is remarkably efficacious in rendering them firmer and more comfortable. But this is not always to be procured; and when the flow of saliva, and the soreness of the gums, formed the chief part of the grievance, I have found nothing more generally useful than a gargle made of brandy-and-water, in the proportion of one part of brandy to four or five of water. This last piece of practice I learned from the present apothecary to the Middlesex Hospital; I have tried it over and over again, and I tell it to you as a thing worth remembering. These little points are by no means to be despised. A very fashionable and successful physician, now dead, used sometimes to say when he met others of his brethren in consultation, 'It is all very well to speculate about the exact situation and the precise nature of the disorder, but the question with me is, "what is good for this, that, or t'other thing?"' A wise physician will seek to combine with an accurate knowledge of disease and settled principles of treatment, those practical expedients and minor appliances which are picked up by casual experience; which could never have been reasoned out, and which sometimes constitute nearly all that we can do for our patient's benefit." (pp. 232-3.)

The concluding paragraph in the above extract contains the statement of a plain but important truth of wide applicability in practice. It is a specimen of that wisdom of common-sense with which we have said these lectures abound.

Ten lectures (xxi to xxxi) are devoted to the more formal and important diseases of the brain and spinal marrow; and in the succeeding seven (xxxii to xxxix) are considered the many analogous affections, which are referrible to functional disorder of some part or parts of the nervous system, &c., &c. We look upon the history of this great family of diseases, both in a pathological and practical point of view, as one of the most valuable portions of the work. An able exposition is given of the difficulties that beset the study of the diseases of the nervous system. We extract a portion of this:

"We often fail to discover *any* deviation from the natural condition of these nervous centres, or of their appendages, even when the disorder of their functions has been broadly displayed. We are not to infer from this that no change has taken place in these parts. The only legitimate conclusion is, that the nervous functions are liable to be deranged, impaired, or suspended by altered conditions, not traceable by our senses or at least not yet discovered by us, of the organs which minister to those functions.

"There may be only one such undiscovered disturbing cause, variable in degree in different cases; or (what is more probable) there may be several such conditions differing in kind. A blow or fall which *jars* the brain, a sudden mental emotion, an electric shock, a tea-spoonful of prussic acid, any one of these causes may destroy life, yet leave no vestige of its action in the nervous substance upon which it operates. It is probable that the fatal condition is not in each case the same.

"We may even form a reasonable conjecture of the manner in which the invisible changes are sometimes brought about. We can conceive, for example, that *undue pressure* upon the nervous pulp on the one hand, or *insufficient pressure* on the other, may constitute conditions of the kind we are in search of; and I shall be able, I think, to convince you that such is sometimes the case. Again, we can conceive that such conditions may be furnished by the varying state of the cerebral circulation. In point of fact, we *know* of some changes in the circulation through the brain which have the effect invariably, first, of modifying, and at length, if they are continued, of arresting, the cerebral functions. If *no* blood be sent through the arteries of the brain, death in the way of *syncope* ensues; if *venous* blood circulates in those vessels, it leads to death by *coma*.

"But whatever may be the nature of the unknown, and perhaps fugitive, physical conditions of the nervous centres, thus capable of disturbing or abolishing their functions, it is useful to keep in our minds a distinct and clear conception of the fact that there must be some such physical conditions. By steadily retaining this idea of their real existence, we may hope at length to get some insight into their nature; which we are the less likely to obtain if we dwell only on the obvious and visible injuries effected in the nervous substance, associated, as they are apt to be, with so perplexing a diversity of symptoms." (p. 351.)

Dr. Watson adopts the views of Dr. Kellie and Dr. Abercrombie respecting the unalterable fulness of the vascular system of the brain; but contends, with them, that variations of the *relative fulness* of the different classes of vessels, or of the vessels of different parts of the brain, together with the *varying pressure* on the cerebral substance thence and otherwise resulting, may very plausibly account for many of the phenomena of cerebral diseases. We have nowhere seen this hypothesis stated with greater clearness or made more probable. We must, however, refer our readers to the work for a full view of its bearings. The following brief extracts may tempt them to consult it:

"But although the actual quantity of blood in the cerebral vessels may continue the same, it does not follow that the relative quantities contained in the arteries and veins should remain unaltered. . . . . . In a very plethoric condition of the body, the arteries which go towards the head partaking of the general fulness, it is not difficult to conceive that there will be an impulse or effort *tending* to the propulsion of an undue quantity of blood into the arteries *within the cranium;* and, under certain circumstances, actually producing a fuller state of those arteries at the expense of the cerebral veins. On the other hand, any sensible interruption of the return of the blood through the veins will virtually augment that impulse upon the arterial current which arises from the force of the general circulation. It is true that we cannot measure or weigh, so as to compare them together, the actual quantities of arterial and venous blood circulating at any period in the cerebral vessels. We never, therefore, can have any demonstrative proof that the kind of derangement, the alteration of balance that has just been supposed, does really occur; but as it evidently, in the nature of things, may occur, so many physiologists believe that it actually does take place, under various circumstances of disease. And taking for granted not only the possibility, but the positive existence of such a derangement, we are enabled to explain many remarkable circumstances connected with the pathology of the brain which might otherwise be altogether mysterious and inexplicable. We can understand how it may happen that a person shall fall down insensible, become completely comatose, and perish, and yet, on the examination of his brain, there shall be found no trace of inflammation or of softening, neither extravasated blood, nor effused serum, nor any change that our senses are capable of estimating. . . . . . . . But there is another principle by which many of the same derangements that leave no vestige behind them in the corpse may, with *equal or greater probability*, be explained. I mean the principle of *varying pressure* upon the nervous substance. Physiologists say that the cerebral matter is incompressible. Upon what grounds this opinion, (which lies at the bottom of the whole of the foregoing doctrine,) may rest, I know not: but whether the brain be compressible or not—whether, that is, it be or be not reducible by pressure into a smaller compass—it is clearly capable of having different degrees of pressure applied to it, and of being pressed out of its ordinary form. We shall see hereafter that by pressure exercised from within, by the distension of what are called the ventricles of the brain, the convolutions on its surface are sometimes flattened, and the natural furrows between them nearly effaced. Pressure there certainly is in what I shall have to describe to you as *hypertrophy* of the brain. There must be considerable pressure on the nervous pulp when blood is poured out within it from a ruptured artery in a cerebral hemorrhage. But the phenomena noticeable when a portion of the skull has been removed by the trephine, show very clearly that the encephalon sustains pressure from varying states of the circulation during perfect health. The surface of the brain, seen through the circular opening in the bone, is observed to pulsate, and to pulsate with a twofold motion. With every systole of the heart the surface protrudes a little, and it again subsides with the succeeding diastole. This shows that the tension of the arteries produced by every contraction of the ventricles of the heart, exerts a degree of pressure upon the contents of the cranium. . . . . . . . . . It is certain, then, that whether the cerebral pulp yields to it or not, there is a constant alternation of a greater and a less compressing force exerted upon it, during life. . . . . . . . . . It is equally certain that the compressing force may transgress its natural limits in either direction; may be too great or too little. The functions of the nervous centres may be perverted or lost when the pressure becomes excessive or, on the other hand, when the pressure is insufficient. . . . . . The pressure may cause fatal coma, and yet no evidence of its operation be left in the dead brain; in cases of *permanent* disease with *occasional* symptoms, accidental circumstances may from time to time determine an undue amount of compressing force or a deficient amount." (pp. 354-60.)

The account of delirium tremens, in lectures xxiii and xxiv, is excellent. The experienced practitioner will at once acknowledge the fidelity of the following graphic description of it; and we should think the student who reads it attentively cannot fail to recognize the disease the first time he sees it. And yet our own experience, and no doubt that of every old physician, accords with that of Dr. Watson as to the frequency with which this purely nervous affection is confounded with inflammation of the brain and its membranes, to the great injury of the patient and the frequent loss of character of the practitioner:

"You will be summoned to a man who is supposed to be mad, or to have brain fever. You find him with a red face, perhaps, and injected eyes, talking wildly and incessantly, fidgeting with his hands, affected often with tremors of the limbs, having a rapid pulse, and bathed in sweat. Now it is very natural that a person not on his guard should look upon these symptoms as indicating inflammation within the head. But if you look closely into the matter you will find in the state of the patient and in his history, some things very peculiar. The delirium you will generally find to be not a fierce or mischievous delirium, but a *busy* delirium: he does whatever you desire him to do, but he does it in a hurried manner, with a sort of anxiety to perform it properly. During the approach of the malady, while he is yet able to go about, he manifests great impatience of any interference, or advice, or assistance in his ordinary duties, which he sets about in a bustling and blundering manner. His loquacity is extreme, and he refers to matters that are not present before him; he is not altogether inattentive to the objects and proceedings that are going on around him, but his mind wanders away to other subjects. There is an odd mixture of the real and the ideal in his thoughts and language. Sometimes he is very suspicious that those who are about him intend him some injury; or that he is surrounded by enemies. You will find also that he does not sleep; that he has not slept perhaps for several nights, but been restless and rambling: and you will generally learn that he has been habitually intemperate, or subject to some great source of care, or anxiety, or excitement: and in many cases he has recently been somehow or other debarred from his customary stimulus. In addition to these points in his history, you will frequently be told that having been unwell, first he has been kept upon low diet, and then, as the delirium came on, he has been freely bled; and that he has been none the better, but commonly worse, for the bleeding. When you gather such particulars as these from his friends (for upon his own statements you cannot place any reliance,) and when you find the delirium to have the characters I have been attempting to describe, and especially when there has been obstinate watchfulness, and the tongue is moist, and the skin is sweating, you may be pretty certain that your patient is affected, not with inflammation of the brain, but with delirium tremens; and that if you bleed him further you will make him worse." (pp. 38-9.)

We must refer to the work for the treatment of this disease. Although Dr. Watson agrees with all practitioners of experience, that opium, in some form or other, is the grand remedy; yet he properly warns his readers that it is not to be given indiscriminately, or trusted to alone. In some cases it must be administered cautiously, if at all; in others it must be combined with depletion in some form; and in others, again, with strong drinks; while, generally, it must be combined with nourishment. In the most common class of cases the following treatment is certainly that which will be found most effectual:

"The opium must be given in full doses; and it must be fearlessly repeated if its desired effect does not follow. If the patients pass many nights without sleep, they will die. I have tried various forms of opium; and I am quite satisfied with morphia. Some persons, however, have not found it so successful as solid opium, or as the common tincture, laudanum. You may try the one or the other, or the one after the other, if you please. No particular rules can be laid down that will suit all cases. After clearing out the bowels by a moderate purgative, you may give three grains of solid opium; and if the patient show no inclination to sleep after two or three hours have elapsed, you may begin to give one grain every hour till he does sleep. Or you may prescribe corresponding quantities of the acetate or muriate of morphia, or of laudanum, or of the black drop, or of Battley's sedative liquor. His room, meanwhile, should be kept dark and quiet. If he sleeps for some time he will awake calmer and more sensible, perhaps perfectly so; and you must withhold the remedy, or continue it in smaller or less frequent doses, according to the circumstances of the case. . . . . . Sometimes this opiate treatment alone is quite enough; sometimes it is not. You will meet with patients who resist very large doses of the drug; but who presently sleep or become composed if you give some of their accustomed stimulus with it,—'a hair (as the vulgar saying goes) of the dog that bit them;' if you put their opiate dose into a glass of gin or a pint of porter. Nervous *exhaustion* goes along with and augments the nervous irritability. . . . . If you learn that, notwithstanding the intemperate habits of the patient, his appetite for food has continued unimpaired and his digestion sound, you will, I believe, generally find that good nourishing diet, strong broths, for example, and the opium, will suffice for the cure. But if the powers and natural sensations of the stomach have been injured and perverted, as is too often the fact, then a temporary recurrence to the habitual stimulus will frequently be necessary; and it is well to ascertain in such cases what the stimulus has been, whether spirits, or beer, or wine, and to order it accordingly. Of course this is not to be continued after the patient has recovered from his delirium." (p. 395.)

In lectures xxvii and xxviii we have an excellent account of the inflammatory and structural diseases of the spinal cord,—a class of cases which may be said to have only been wrought out as definite morbid states in recent times. The author most justly observes, that "the structural diseases of the spinal cord will most clearly reveal themselves by their symptoms to him who most distinctly perceives, and most accurately bears in mind, the *physiology* of that part of the nervous system;" and for this reason he very judiciously prefaces his account of them by a brief but explicit account of the anatomy and physiology of the spinal marrow. (pp. 554-8.)

Returning to the subject of strictly cerebral diseases, the author enters upon the consideration of apoplexy in lecture xxviii, and devotes the greater part of four lectures to it. This portion of the work is rendered extremely interesting by the relation of many striking cases. We had marked numerous passages for extract, but we must content ourselves with a very few specimens. The young practitioner, fresh from the schools and full of the physiology of the nervous system and of the pathological anatomy of the brain, may think it unlikely that he should ever confound real apoplexy with simple coma from mere temporary causes, as from drunkenness; and yet he will find the diagnosis not always easy in practice. So, at least, Dr. Watson says; and so we ourselves have found it on more than one occasion.

"If you were summoned to a person in the comatose state I have been describing, how could you tell whether he was affected with apoplexy or labouring under the influence of a large dose of opium, or merely dead drunk? Why, so far as the cerebral functions are concerned, you cannot discriminate the one from the other. In each case there is profound coma; but the cause of the coma is different in each, and you must seek to ascertain that cause in the history and other circumstances of the patient: you inquire whether he is known to have been drinking, you try if you can perceive the odour of wine or of spirits in his breath; or you endeavour to make out whether he has been low-spirited or in known difficulties; in short, whether it is likely he may have swallowed poison. But from the actual condition of his sensorial functions, you cannot solve the question." (p. 470.)

In the two following cases, related in illustration, the proper diagnosis was made; but we quote them as instances of a condition which we have known mistaken for apoplexy. The second case is an example of the happy and amusing manner in which Dr. Watson so often illustrates his subject:

"A man was found lying in Smithfield in a state of total insensibility, and motionless, except that he still breathed. He was carried into St. Bartholomew's Hospital. The house-surgeon thought he smelt the smell of gin in his mouth, and thereupon very properly made use of the stomach-pnmp; by means of it he discharged a large quantity of ardent spirit; and in the course of a few minutes the man revived, shook his ears, and walked away. If the gin had been suffered to remain in the stomach, and if the remedies of apoplexy had been vigorously put in force, the absorption of the poison would have been thereby accelerated; and the debauch would probably have had a fatal termination. . . . The father of the late professor James Gregory of Edinburgh (who used to relate the case in his lectures,) was once called out very late in the evening to visit an old gentleman of that place. He found him in a completely comatose condition, his wife crying, and his household all plunged in grief and distress. They told him that the patient, whom he now saw in a fit, had come home, and upon the servant's opening the door to him, had fallen into the passage, on his back, in a state of insensibility. Dr. Gregory learned, however, that he had been at the 'club,' and he knew well enough that this club was composed of choice spirits, fond of their cups, although the gentleman's wife did not know as much. Therefore he ventured to express his 'hopes' to the wife that her husband was drunk: a very charitable view of the case, at which she was extremely affronted and indignant. He persisted, however, in his opinion, and not long afterwards the patient began to recover his senses. It turned out that he had partaken more liberally than the rest of the club, and was the *first* to be intoxicated. Two of his companions carried him home quite incapable of motion; but not liking to introduce him themselves to his wife in that predicament, they placed him with his back against the door, rang the bell, and decamped. Of course when the servant came to open the door, his master tumbled senseless on the floor. I need not point out to you the ridicule which the physician would have brought upon himself, and the damage he might have inflicted upon his patient had he busily applied, in this case, the ordinary remedies of apoplexy." (pp. 470-1.)

It has long been received among physicians as an almost settled point in pathology, that hypertrophy of the heart is a common exciting cause of apoplexy. Dr. Watson denies this as a general truth, and gives numerous strong reasons for considering the two events as mere coincidences, and not standing in the relation of cause and effect. (pp. 515-7.) Dr. Watson's views on this point are held by others. (See our Review of Canstatt in the present Number, p. 111.)

As usual, the treatment of this disease inculcated by Dr. Watson is discriminating, and running into neither extreme. The following graphic illustration of "active practice," so much in vogue with some practitioners in this disease, contains a lesson of most grave import:

"Practitioners are too apt, in this, as in other instances, to be guided in their choice of remedies by the *name* of the disease, and to treat all cases of apoplexy alike. I remember being much amused by the perplexity which a friend of mine once told me he had felt on being summoned by letter many miles into the country to see a gentleman who had been struck with apoplexy. As he posted down, he earnestly revolved in his mind what he might be able to advise when he should reach the house of sickness. He felt confident that the patient must already have been copiously bled, cupped or leeched, blistered, and thoroughly dosed with calomel, senna, and croton oil. Mustard poultices had doubtless been applied to his legs. My friend was distressed to think that while much would be expected, nothing would be left for him to do worthy of so long a journey, and so heavy an expense to his client. A clyster of turpentine might yet, perhaps, be an untried expedient. His cogitations were cut short, however, and his cares relieved, by an express which met him half-way on the road to announce that the patient was dead." (pp. 519-20.)

The important practical question respecting bloodletting in apoplexy is pretty fully discussed. Although Dr. Watson's practice is, in this respect, very discriminating, we are not sure but some of his readers will consider him as leaning rather too much to the side of depletion. At the present time, however, there is some danger lest the ill effects of indiscriminate venesection, now so fully recognized, may not lead to a practice as dangerous in the opposite extreme. The following extracts show that our author can give good reasons for his treatment, whether it is to bleed or to refrain from bleeding:

"If the pulse be full, hard, or thrilling, (sometimes it feels like a tense vibrating rope,) or if there be obvious external signs of plethora of the head, you must extract blood. You are not to refrain from bleeding the patient because he is pale, if the pulse warrants it; nor may you omit taking blood if the head and face be turgid, although the pulse be small, for that smallness may depend upon organic disease of the heart. On the contrary, if his skin is pale and cold, and his pulse feeble and flickering, you would probably ensure your patient's death, or determine the accession of palsy, if you withdrew from the failing heart and blood-vessels a portion of their natural stimulus. . . . . . . If the patient to whom you are summoned be stupid and drowsy rather than faint, and his pulse and appearance warrant the conclusion of plethora capitis, the first thing to be done is to place him in a semirecumbent position, with his head raised; to loosen any tight parts of his dress, especially his neckcloth and shirt-collar, and whatever might press upon the *neck;* and then as quickly as possible to bleed him from the arm. We know that in some cases the apoplectic state occurs when as yet no injury has been done to the brain—no effusion, no laceration of its texture—and we may hope, by timely and vigorous measures, to *prevent* these terrible evils. We never can be sure that there is blood extravasated in such cases, and we must act, in the first instance, upon the presumption that there is not. We are especially encouraged to take away a considerable quantity of blood by venesection when we perceive external signs that the vessels of the head are full, redness and turgescence of the face, throbbing and prominence of the temporal arteries, distension of the superficial veins of the neck and forehead. Our object is to take off the strain upon the internal vessels by bleeding in such a manner and to such an amount as shall produce a decided effect upon the general circulation. . . . . . Again, the whole state of the patient may approximate more or less nearly to the state of syncope; the pulse being weak, the aspect pinched and bloodless, and the skin cool. In this condition no good, but the contrary, is to be expected from bloodletting of any kind. You will do better to apply warmth, cautiously, to the surface, and cautiously to administer what are called diffusible stimuli, of which the preparations of ammonia afford the most eligible forms. Five grains of the sesquicarbonate, or half a drachm of sal volatile, mixed with camphor, julep, are ordinary doses. Stand by till the first stunning effect of the internal shock passes off; and carefully watch meanwhile for symptoms of reaction." (pp. 520-3.)

Of the propriety of purging there is much less difference of opinion; and there is little risk in being too active here:

"Purgative medicines are of signal service in apoplexy. They empty the intestines, which are oftentimes loaded, and which, by distending the abdomen, have occasioned, perhaps, undue pressure against the diaphragm, embarrassed the breathing, and through it the cerebral circulation. Another very important purpose of hard purging, which I have frequently pointed out before, is the producing of copious watery discharges from the bowels, whereby the blood-vessels are drained, and the tendency of blood to the head especially relieved. If the patient can still swallow, you may give him half a scruple of calomel, and follow it by a black dose. If the power of deglutition be lost, the croton oil becomes a most valuable remedy. Dr. Abercrombie suggests that it may be conveniently introduced into the stomach, suspended in thick gruel or mucilage, by means of an elastic gum-tube. But really this is not necessary. If two or three drops of the oil be put upon the tongue, as far back as possible, it will produce its specific effect very readily and well. But we are not to wait for the operation of aperients given by the mouth. Strong purgative and stimulating enemata must be thrown into the rectum; half an ounce or six drachms of turpentine suspended, by the help of the yelk of an egg, in gruel or warm water. We very often witness decided signs of amendment upon the free operation of a purgative." (pp. 523-4.)

In the thirty-first and thirty-second lectures we have a very full and interesting account of the various kinds of *local paralysis*, more especially of the most common and important form, *facial palsy*. There are few diseases which are more calculated to exhibit to patients the pathological and physiological knowledge of the physician than this; and therefore it is one that it is especially the interest of the young practitioner to study. Before the full light of physiology was thrown on it by Sir Charles Bell, it was very apt to be considered as having its origin, like other forms of paralysis, within the skull; and patients were often treated very improperly in consequence. The danger, at present, perhaps lies on the other side. The affection is now so well known as to be taught in all the schools; and it will probably be easily recognized by most men when first met with in practice. It may, however, not always be remembered, that the same external phenomena sometimes acknowledge an internal cause precisely similar in kind to that of hemiplegia. Cases of this kind are extremely perplexing; and it behoves the young practitioner well to weigh all the symptoms of each individual case before he delivers a positive diagnosis and prognosis. The present work will afford him most important assistance towards the establishment of correct views. The following case offers one of the best illustrations we have met with, of the kind of cases now under consideration; its features of danger, though sufficiently obvious to a man of experience, as they were to Dr. Watson, might very readily have been overlooked by a young physician:

"The following case caused me much anxiety, for the subject of it was a personal friend of mine. I was summoned to his house in the autumn of 1829, and found him with complete palsy of the left side of the face. It had existed a day or two. I shall not describe the appearances and symptoms that resulted from the paralysis; for they were precisely the same as were presented by the girl Smith; [a case of purely local facial palsy;] and they are always, and necessarily, very much alike. But though the *palsy* was strictly limited to this set of muscles, there were other symptoms present which indicated that the interruption of the functions of the portio dura was connected with some morbid condition within the cranium; nausea and vomiting, twitching of the muscles of the *other* side of the face, great drowsiness, and a slow pulse, forty-eight only in the minute. He lurched also, and staggered as he walked; but he distinguished this from the reeling of the vertigo, and denied the latter sensation altogether. He was deaf, too, on the affected side.

"His previous history did not tend to diminish the fears which his actual state excited. In the previous February he had been attacked rather suddenly, with intense pain just above the right eyebrow, and became extremely drowsy. Being desirous, on account of those feelings, to excuse himself from a dinner engagement, he found that he was unable to write a proper note: he could not remember how he ought to express himself. All these symptoms soon passed off, after the operation, I believe, of an emetic. But he had another attack of the same kind in the subsequent May—the same severe pain over the right brow, with great drowsiness and confusion of mind. He could not recollect the first line of the Æneid. He wished a friend to look at the *signatures* of some letters that arrived; and though he knew the root, he could not tell how the word he wished to use was formed—whether it was signition, or signation, or signature. The digestive organs on this occasion were made the object of treatment, and he soon got well. There was another instructive part of his history, and therefore I mention it. Before these attacks he was in the habit of eating and drinking freely, and his power of digestion was supposed to be enormous. After the attack in May he commenced a strict course of temperance. He drank no wine *till three or four days before* the occurrence of the facial palsy ; he had then taken it again, and had about four glasses daily, and on one of the days he drank two glasses of champagne.

"It was of some moment to this gentleman, not only that he should recover, but that he should recover quickly. He had been appointed by government to a mission to Ceylon, and all his equipment was already on board a vessel, which would sail in a fortnight. Cupping behind the ears, blistering, purgatives, and small doses of calomel continued till the gums were slightly sore, removed the paralysis and all the other symptoms, in about ten days. He went to Ceylon, and performed his mission so ably that after his return the government appointed him to one of far greater importance in India, where he now is. He has remained perfectly well, and possesses one of the clearest and strongest intellects that I am acquainted with." (pp. 542-3.)

The next eight lectures (xxxii to xxxix) are devoted to tetanus, hydrophobia, epilepsy, chorea, hysteria, and neuralgia ; and are full of interest of every kind. But here our readers must cater for themselves : we guarantee them from disappointment. From among the many passages marked by us for quotation, we will select part of one on the diagnosis of epilepsy and hysteria, because this also is a point which is very apt to puzzle and distress young physicians ; and not without good cause, as Dr. Watson testifies :

"It is of great importance to be able to render the diagnosis certain and accurate. It is a dreadful announcement to have to make to a father or a mother that their child is epileptic ; whereas hysteria, though it is sufficiently distressing, is attended, in nine hundred and ninety-nine cases out of a thousand, with no ultimate peril either to mind or body. In some instances the diagnosis is perfectly easy, in others it is dubious and full of anxiety. Whenever you fail to satisfy yourselves completely as to the nature of a given case, you will do well, in legal phrase, to give your patient the benefit of your doubt, and acquit her of epilepsy, or pronounce her guilty of the minor offence of hysteria." (pp. 668-9.)

The following extract gives some of the more important marks of distinction during the convulsive paroxysm :

"In the epileptic *fit* there is an entire loss of consciousness. The patient, on emerging from the paroxysm, recollects nothing of what has been going on during its continuance. It is not so in the hysterical fit, the loss of consciousness is very seldom complete, and it never occurs at the outset of the attack. The patient often is able to repeat, (though she may not always choose to confess it,) what has been said by the bystanders during the period when she seemed insensible. This is a point of distinction well worth remembering, for more reasons than one. It not only helps the diagnosis when the fact comes out, but it suggests certain cautions to ourselves. We must take care not to say anything by the bedside of an hysterical patient which we do not wish her to hear ; and we may take advantage of her apparent unconsciousness, and pretend to believe in it, and speak of certain modes of treatment which she will not much approve of, but the very mention of which may serve to bring her out of the fit.

"In the epileptic paroxysm the face is usually livid ; and foam, which is frothy with air or red with blood, escapes from the patient's mouth. These are symptoms which we do not see in the fits of hysteria. The convulsive movements even offer some characteristic shades of distinction. In epilepsy they are often more marked on one side of the body than on the other, and less irregular ; the same movements are rapidly repeated ; there is a strangling rattling in the breathing ; while in hysteria the forcible flexion and extension of the limbs and the contortions of the trunk are more sudden and, as it were, capricious ; the respiration is deep, sighing, mixed with cries and sobs and often with laughter. But perhaps the convulsive motions differ most in the face ; the epileptic expression is usually frightful ; the eyelids half open, the eyeballs rolling, the mouth drawn to one side, the teeth grinding, the gums exposed by the retraction of the lips, the tongue protruded and bleeding, the complexion leaden: while in hysteria the cheeks are red, but at rest; the eyelids are closed and trembling; if you raise the upper one you will see the eye fixed, perhaps. but it is bright, and very different from that of the epileptic, which, if it be not rolling, is dull, projecting, and the pupil usually dilated." (pp. 669-70.)

The diseases mimicked by hysteria are not overlooked by our author; and the young practitioner will perhaps be surprised to find among the number some inflammatory affections which he may think it impossible to be aped sufficiently well to deceive him. Let him, however, be not over confident. The following paragraph recalls to our recollection, after a period of some five and twenty years, the case of a young lady, which we maintained to be peritonitis, against an experienced apothecary with whom we were called to consult. In our pathological zeal, in those early days, we would have almost suffered martyrdom in support of our opinion; but, alas, in a day or two the hysteric tympany, for such it was, suddenly disappeared, and carried away our school-pathology and our comfort for the time along with it. We have seen many such cases since; but as our knowledge of actual disease augmented, our confidence in nosological definitions, and eke our alacrity for pathological martyrdom, abated in like proportion.

"One of the diseases which is most often copied by hysteria, is *inflammation of the peritoneum.* You will find a patient complaining of acute pain of the abdomen, aggravated by the slightest pressure; and she shall have, perhaps, a hot skin, a quick pulse, and a furred tongue. When you meet with such symptoms in a young female in whom there is any derangement or irregularity in the uterine functions, you will do well, before you bleed her to syncope and cover her abdomen with leeches, to ask yourselves whether all this suffering may not be simply nervous. Search into her previous history as narrowly as you can; if you find that she has had similar attacks before; if she has been known to suffer hysterical fits; and if the tenderness is excessive and, as it were, superficial, felt upon the slightest touch as much as when the firmer pressure is made, you may generally spare the bloodletting, purge the patient well, and cause an assafetida enema to be thrown into the rectum; and in a few hours you will find that the peritonitis has vanished." (p. 672.)

But having got nearly to the end of the first volume of our book, we must hold our hand. Our readers must find out for themselves what the second contains. We will only say, that both are from the same mint; and the last has the same ring of true metal as the first. We hope, however, by what we have done, we have shown ourselves qualified at least for the membership of the school of criticism named in the beginning of our article, and whose unsavory title we would rather not repeat. If we do not join it yet, it may perhaps only be because we are foolishly prejudiced in favour of our own; or because, like other great men, we have our idols and strange gods that seduce us from the true faith. This, however, we will say, that if we thought ourselves likely to be often tempted by such works as Dr. Watson's, we should feel less confident in maintaining our old practice, however we might continue to profess our old principles. But we shrewdly suspect that we are not in much danger of being often so tried. Dr. Watson is too busy with his patients to write us another book soon; and we shall therefore probably go on quietly in the old track, as before,—until again, at some distant day, led into temptation either by him or by some other wise charmer with voice potential as his own.

